# Comparing the Effect of Different Antibiotics in Frozen-Thawed Ram Sperm: Is It Possible to Avoid Their Addition?

**DOI:** 10.3389/fvets.2021.656937

**Published:** 2021-05-07

**Authors:** Luis Anel-Lopez, Marta F. Riesco, Rafael Montes-Garrido, Marta Neila-Montero, Juan C. Boixo, César Chamorro, Cristina Ortega-Ferrusola, Ana Carvajal, Jose R. Altonaga, Paulino de Paz, Mercedes Alvarez, Luis Anel

**Affiliations:** ^1^Investigación en Técnicas de Reproducción Asistida - Universidad de León, Instituto de Desarrollo Ganadero y Sanidad Animal, University of León, León, Spain; ^2^Department of Veterinary Medicine, Surgery and Anatomy, University of León, León, Spain; ^3^Cellular Biology, Department of Molecular Biology, University of León, León, Spain; ^4^Laboratory of Equine Reproduction and Equine Spermatology, Veterinary Teaching Hospital, University of Extremadura, Cáceres, Spain; ^5^Department of Animal Health, Facultad de Veterinaria, Universidad de León, León, Spain

**Keywords:** ovine semen, artificial insemination, antibiotics, fertility, sex-ratio, prolificacy, antibiotic resistance

## Abstract

It is crucial to perform a deep study about the most extensively used antibiotics in sperm extenders. Most of the protocols and concentrations used in ram are direct extrapolations from other species. It is important to establish species-specific antibiotic treatments to optimize their use and if it is possible to reduce the quantity. Previews studies have assessed some aspects of sperm quality *in vitro*, but this study aimed to go further and assess the effect of three different antibiotic treatments, which are the most extensively used, not only in sperm quality or assessing the inhibitory effect on bacterial growth but also assessing these important parameters of productivity such as fertility, prolificacy, fecundity, and sex-ratio during a freeze-thaw process. Gentamicyn (G) treatment showed the worst results, not only concerning sperm quality but also in the reproductive trials exhibiting a toxical effect at the experiment concentration, and being the most powerful inhibiting bacterial growth. For its part, Lincomicyn-spectinomycin (LS) showed similar results inhibiting bacterial growth but it did not show a detrimental effect either in sperm quality or in reproductive parameters. Penicillin-streptomycin (PS) showed good results in the sperm quality and in the reproductive *in vivo* trials, but it showed a very poor effect inhibiting bacterial growth probably due to some kind of antibiotic resistance. According to our results, there is not a significant positive relationship between the higher bacterial inhibitory activity of LS and PS samples, and the sperm quality respect Control samples (without antibiotics). In the case of G, which exhibited the most effective as antibacterial, we observed a toxic effect on sperm quality that could be translated on productivity parameters. Our results suggest that the bacterial contamination control in frozen-thawed semen may be possible without the use of antibiotics, although the effects of longer periods of cooling storage and different temperatures of storage need to be further investigated for animal semen. At this point, a reflection about a drastic reduction in the use of antibiotic treatments in sperm cryopreservation is mandatory, since freezing conditions could keep sperm doses contamination within the levels recommended by regulatory health agencies.

## Introduction

Semen collection and sperm manipulation are not sterile processes, and bacterial contamination, especially environmental and non-pathological but also pathological species, of sperm samples cannot be avoided (1, 2). In ram, the final ejaculate collected is usually contaminated at least with bacteria from the technician, artificial vagina, penis, and prepuce. In this way, bacterial contamination could affect not only the sperm quality but also the final yield of the production. The bacteria-sperm interaction has been widely studied, especially in human sperm with *E. coli* as a model of contamination concluding that the spermicidal effect is concentration-dependent (3). Several negative effects have been reported from the bacterial contamination on the sperm quality such as the sperm motility impaired by bacterial adhesion and agglutination (4, 5), inducing morphological changes (6) altering the sperm function, increasing the phosphatidylserine translocation and the apoptosis activation (7). In addition, some studies suggest that bacterial contamination may increase the antibodies production affecting the glycocalyx complex in the sperm surface (3, 8). To prevent disease transmission in most domestic species, the addition of antibiotics to sperm extenders is mandatory in the European Union and so common in other countries outside (9). In this context, the antibiotic supplementation as an additive in most of the handling and preservative sperm extenders has been widely used in many species; in domestic ones such as ram (10), bull (11), stallion (12), or boar (13) and wild species: red deer (14) or brown bear (15). Many studies have demonstrated the beneficial effect of antibiotics inhibiting the bacterial proliferation in the sperm samples during their storage (1, 16–18). Most of these studies have been carried out in different species than ram, without species specific studies, which could generate unexpected effects on relevant production aspects such as fertility, prolificacy, fecundity, or sex-ratio. In the same way, most of these studies have been focused on the assessment of the effect of the antibiotics on the sperm quality assessed *in vitro* in buffalo, bull, and ram (10, 11, 19). It is important to take into account that the use of sperm insemination doses with contamination could be related to deleterious effects on the female reproductive tract and estrus status (20, 21), reducing the embryonic survival, or even reducing the litter size (22) in swine. However, the effects of antibiotics in important productive parameters such as fertility, prolificity, fecundity, or sex-ratio have not been widely studied in ram, usually being extrapolations and copies from other species. To get a deep knowledge about the effects of antibiotics not only on the sperm quality but also in the future production of the sperm doses is very important to optimize protocols and to reduce the use of antibiotics since the antimicrobial resistance supposes a global and serious danger not only for the human or animal health but also for the economics (23).

This study aimed to go further and assess the effect of three different antibiotic treatments, which are the most extensively used, not only in the sperm quality or assessing the inhibitory effect on the bacterial growth but also assessing these important parameters of productivity such as fertility, multiple lambing frequency, prolificacy, fecundity, and sex-ratio, which have a very important impact on the ovine livestock.

## Materials and Methods

### Reagents and Media

All the products used in this paper were of at least reagent grade and were acquired from Sigma Aldrich (Madrid, Spain) unless otherwise stated. The medium for cytometry assessment was PBS.

### Animals and Sperm Collection

Sperm samples were collected from mature ram males during the breeding season. The ejaculates were collected by artificial vagina at 40°C (Minitüb, Tiefenbach, Germany), and the tubes were maintained at 35°C during the initial evaluation of sperm quality. The volume was calculated using the graduation marks of the collection tubes. Mass motility was assessed by microscopy (warming stage at 38°C, 40x; score: 0–5; Labophot-2, Nikon, Tokyo, Japan), and the sperm concentration was assessed using a Nucleocounter n-100 (DADOS MARCAETC). Only ejaculates with good quality (volume: ≥0.5 mL; mass motility: ≥4; sperm concentration ≥3,000 × 10^6^ mL^−1^) were used and processed for the experiment.

### Experimental Design

Before freezing, samples (1 valid ejaculate per male) were diluted down to a final concentration of 100 × 10^6^ sperm/mL in their respective extender (Tes-Tris-Fructose 20% egg yolk_4% glycerol_320mOsm/Kg) as follows: without antibiotics (Control), with Penicillin-Streptomycin (PS) to a final concentration of 500 UI and 625 μg/mL, respectively, Lincomycin-Spectinomycin (LS) to a final concentration of 300 and 600 μg/mL, respectively, and Gentamicin (G) to a final concentration of 1,000 μg/mL. After this, samples were frozen and thawed as explained in point Sperm Cryopreservation. Samples were assessed *in vitro* just after thawing (T0) and after submitting them to a stress test of 2 h of incubation at 37°C (T2), except for the microbial assessment, which was carried out just after thawing (T0). For the *in vivo* trial (artificial insemination), samples were processed as described in statement Fertility Trials.

### Sperm Cryopreservation

Sperm extended samples were cryopreserved following the protocol (24) modified by Alvarez et al. (25). Samples were refrigerated at −0.25°C/min from 30 to 5°C in the refrigerated chamber. After 2 h of equilibration at 5°C, samples were packed in 0.25 ml straws and then frozen in a programmable biofreezer (Kryo 10 Series III; Planer PLC, Sunbury-on-Thames, UK) at −20°C/min up to −100°C, transferred to liquid nitrogen containers, and stored for a minimum of 1 month. Thawing was performed by dropping straws in the water at 65°C for 6 s. One part of the samples was used to carry out the artificial inseminations; another part of the straws was used to carry out sperm *in vitro* assessment, just after thawing (T0) and after 2 h of incubation at 37°C as a stress test (T2). The last part of the samples was used to perform the microbial culture.

### *In vitro* Sperm Evaluation

Straws from 9 males (one ejaculate per male) were used for the *in vitro* assessment as follows.

#### Computer-Assisted Sperm Analysis

Samples were diluted to 30 × 10^6^ sperm/mL in their freezing extender to check the motility. A warmed Makler counting chamber was loaded with 5 μL of the sample. The analysis was carried out using a CASA system (Computer Assisted Sperm Analysis), consisting of an optical phase-contrast microscope (Nikon Labophot-2) (fitted with negative phase-contrast objectives and a warming stage at 37°C), a Basler A312fc camera (Basler, Germany), and a PC with the sperm Class Analyser software (ISAS v. 1.2; Proiser, Valencia, Spain). The magnification was 100 × . At least five fields per sample were acquired at an acquisition rate of 25 images per second, recording a total of 200 motile sperm. The following parameters were used for the study: total motility (%; TM), progressive motility (%; PM), average path velocity (μm/sec; VAP) straight-line (rectilinear) velocity (μm/s; VSL), and amplitude of lateral head displacement (μm; AHL).

Image sequences were saved and analyzed afterward using the editing facilities provided by ISAS. Sperm were considered motile when VCL > 10 μm/s and progressive if VCL >10 and straightness (STR) >80%. The progressive sperm subpopulations were classified according to velocities as follows: Slow (VCL <25), Medium (VCL >25 and <65), and Rapid (VCL >65). Other events different from spermatozoa were removed, and settings were adjusted in each case to assure a correct track analysis.

#### Flow Cytometry

Flow cytometry acquisition was performed in a flow cytometer (MACSQuant Analyser 10, Miltenyi Biotech, Madrid, Spain) equipped with three lasers emitting at 405, 488, and 635 nm and 10 photomultiplier tubes (PMTs): V1 (excitation 405 nm, emission 450/50 nm), V2 (excitation 405 nm, emission 525/50 nm), B1 (excitation 488 nm, emission 525/50 nm), B2 (excitation 488 nm, emission 585/40 nm), B3 (excitation 488 nm, emission 655–730 nm; 655LP + split 730), B4 (excitation 499 nm, emission 750 LP), R1 (excitation 635 nm, emission 655–730 nm; 655LP+split 730), and R2 (excitation 635 nm, emission filter 750 LP). The system was controlled using MACS Quantify software (Miltenyi Biotech, Madrid, Spain). These excitation and emission wavelengths allowed us to find probe combinations that can simultaneously assess multiple parameters in a large number of sperm (a total of 40,000 events per sample and at least 20,000 sperm cells, at a flow rate of 200–300 cells per second, were acquired). Data were analyzed using FlowJo v.10.2 (Ashland, USA).

#### Simultaneous Flow Cytometric Assessment of the Viability, Caspase 3 and 7 Activity, and Metabolic Activity (ROS Generation)

Sperm samples of different experimental groups were diluted in PBS medium to obtain a total of 2 × 10^6^ of sperm per sample; these samples were washed and centrifuged at 500 g for 10 min at room temperature (RT). Lyophilized Zombie Violet™ (Biolegend, San Diego, California, EEUU) dye was reconstituted in DMSO following the manufacturer's instructions (100 μl of DMSO to one vial of Zombie Violet™ dye). CellEvent™ Caspase-3/7 and CellROX™ Deep Red (Invitrogen, Eugene, Oregon, EEUU) were purchased as a 2 mM and 2.5 mM stabilized solution, respectively. Stock solutions of fluorescence probes were prepared at 1 μL and kept at −20°C in the dark until needed.

Zombie Violet™ stock solutions were resuspended in 1 mL of PBS while CellEvent™ Caspase-3/7 and CellROX™ in 10 μL. After samples centrifugation, the supernatant was discarded, and the sperm pellet was incubated at RT for 30 min in the dark with 96 μL of Zombie Violet™ (membrane integrity probe) (1:1,000 final dilution), 2 μL of CellEvent™ Caspase-3/7 (apoptosis marker) 4 μM final concentration, and 2 μL of CellROX® (ROS content labeling) 5 μM final concentration. After that, another washing step was performed to stop cell staining, and the pellet was resuspended in 1 mL of PBS, carrying out the analysis immediately by flow cytometry.

The interest sperm subpopulations assessed were plotted as follows: Non-Apoptotic Viable Sperm (Zombie low intensity, Caspase 3&7 negative), Apoptotic Sperm (Zombie low intensity -alive-, Caspase 3&7 positive), and High Metabolically Active Sperm (Zombie low intensity, CellROX positive).

### Bacteriological Assessment

Straws from 8 males for each treatment, without antibiotics (control), with Penicillin-Streptomycin, with Lincomycin-Spectinomycin, or with Gentamicin, were thawed and 150 μL of each sample were plated onto blood agar plates (Oxoid, Wesel, Germany). The inoculum was spread rapidly over the entire agar surface with a sterile Digralsky spreader. Plates were incubated at 37°C under aerobic conditions and inspected after 48 h incubation. Bacterial growth was expressed as colony-forming units CFU/mL. Subcultures were performed until pure cultures were obtained. Primary identification was based on Gram staining and catalase and oxidase tests while confirmation was carried out using matrix-assisted laser desorption ionization time-of-flight mass spectrometry (MALDI-TOF MS, Bruker, Madrid, Spain).

### Fertility Trials

For the fertility trial, sperm doses (25 x 10^6^ sperm/straw), frozen-thawed as above described (2.4), from 10 mature males (Churra breed) were used. The experimental samples were randomly and sequentially distributed through 7 commercial farms following a commercial artificial insemination program (Churra breed improvement program) under the strict supervision of our research group. Adult Churra ewes (852 females between 2 and 5 years old lambed previously) were subjected to treatment for estrous induction and synchronization using intravaginal sponges with 20 mg fluorogestone acetate (Chronogest®, MSD) over 14 days. The sponges were removed and 500 IU of eCG were injected -IM- (Folligon®, MSD). Laparoscopic inseminations were performed by two vets with extensive experience, between 64 and 67 h after the removal of the sponges. The animals, having fasted for the previous 24 h, were tied to a special cradle (IMV), placed on an inclined plane (45°), and the area in front of the teat was shaved and cleaned. Local anesthesia (mepivacaine HCL 2%, Braun^TM^) was applied to the puncture points. Then two portals (for vision and manipulation/injection) were inserted by performing a pneumoperitoneum (CO_2_). The semen, placed in a special applicator (Transcap®, IMV), was injected under visual inspection into each uterine horn (0.12 mL, 12.5 × 10^6^ spz). Fertility [(lambing ewes/inseminated ewes) × 100] was calculated according to the births registered at 137–154 days post-insemination. Moreover, viable offspring and sex were registered, and prolificacy (lambs/lambed ewes), multiple lambing frequency [(multiple lambing/total lambing) × 100], fecundity (lambs/inseminated ewes) and sex-ratio [(female lambs/total lambs) × 100] were calculated.

### Statistical Analysis

For the *in vitro* quality, data were analyzed using the SAS™ V.9.1 Package (SAS Institute Inc., Cary, NC, USA). Results are shown as means and standard errors of the mean. The normality of data was verified by Kolmogorov-Smirnov tests. Analyses of the data were carried out using linear mixed-effects models (MIXED procedure, ML method), including the type of antibiotic (C, PS, LS, and G) and incubation time after thawing (0 vs. 2 h) as fixed factors, and males as a random effect. Significant fixed effects were further analyzed using multiplecomparisons of means with Tukey contrasts. A significance level of *P* < 0.05 was used; *P* < 0.1 was considered as a trend.

Fertility, multiple lambing frequency, and sex ratio data were compared using a GENMOD procedure considering a binary response model. The statistical model included the type of antibiotic (C, PS, LS, and G) as a factor, and fertility, multiple lambing frequency, and sex-ratio as a response variable. Between-group differences in the frequency were tested using Wald Chi-Square. For sex-ratio, the study was completed comparing each experimental group to the “expected value” (50:50). Prolificacy and fecundity data were compared using a GLM procedure using the type of antibiotic as a factor; between-group differences were tested by Duncan test. The significance level was set at *P* < 0.05.

## Results

### Sperm Motility

**Figure 1:**
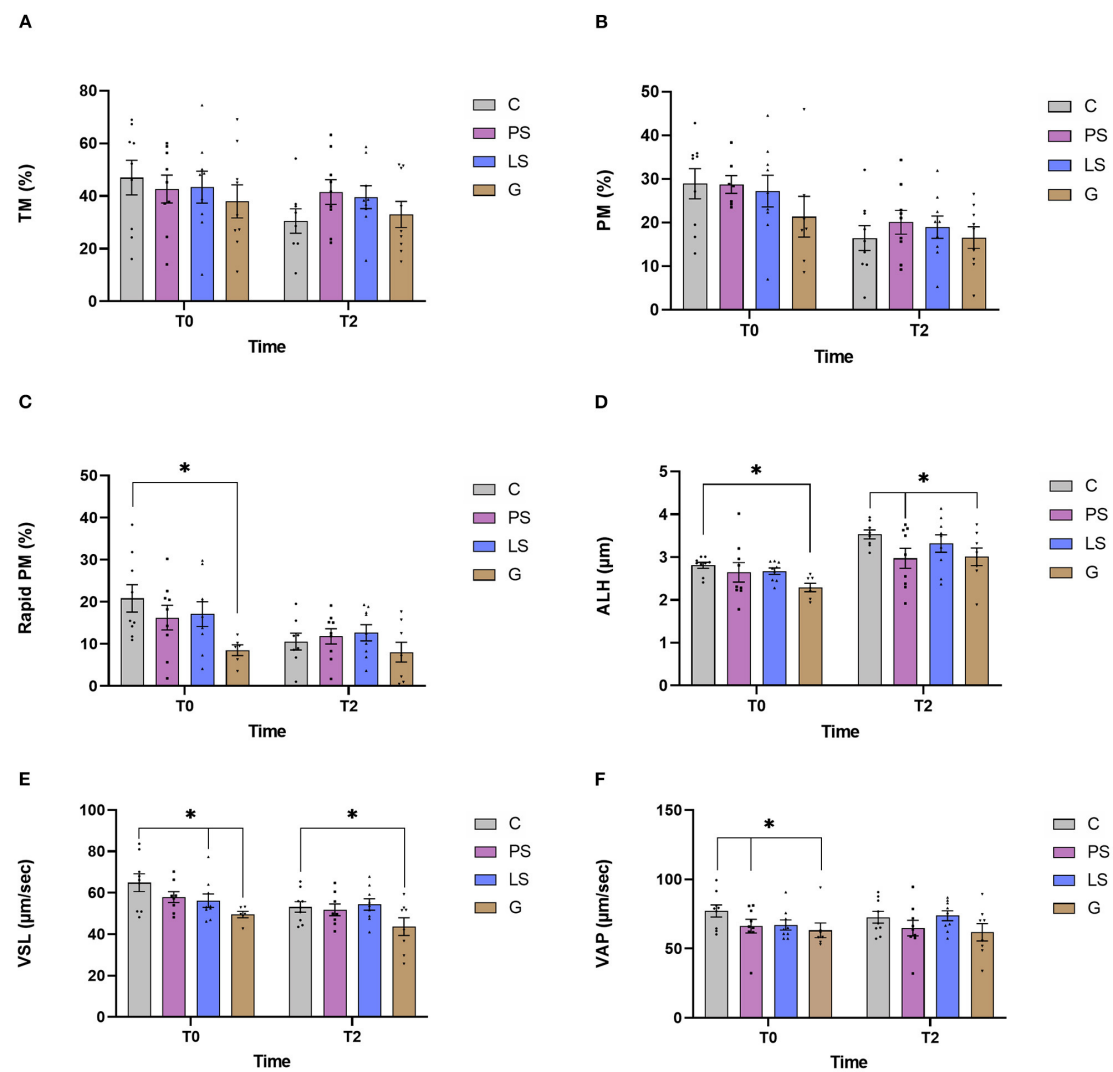
Ram sperm motility data in the four experimental groups by time (just after thawing -T0- and after 2 h of incubation at 37°C -T2-): Control (C), without antibiotics; Penicillin-Streptomycin (PS); (500 UI)−625 μg/mL, respectively; Lincomycin-Spectinomycin (LS); 300–600 μg/mL respectively; and Gentamicin (G) 1,000 μg/mL. **(A)** Total motility (TM, %); **(B)** progressive motility (PM, %); **(C)** rapid progressive motile sperm (Rapid PM, %), **(D)** amplitude of the lateral head movement (ALH, μm), **(E)** velocity according to the straight path (VSL, μm/s), and **(F)** velocity according to the smoothed path (VAP, μm/s). Nine males were analyzed (1 ejaculate per male) including the same males in each experimental group. Graph dots represent individual male ejaculate. Significant differences (*P* < 0.05) are represented with an asterisk between the antibiotic treatment and the Control sample without antibiotics.

The mean, standard error, and male distribution for several sperm motility parameters are showed in Figure 1. Just after thawing non-significant differences (*P* > 0.05) were observed either for TM or for PM among the different antibiotic treatments (Figures 1A,B). After 2 h of incubation at 37°C, all the samples showed a significant decrease from 0 h, but no difference was observed between treatments. In contrast, when assessing the rapid PM, G samples showed significantly lower values (*P* < 0.05) than C just after thawing (Figure 1C). In the same way, when assessing ALH and VSL G samples showed significantly lower values (*P* < 0.05) than the C samples not only just after thawing but also after 2 h of incubation at 37°C (Figures 1D,E). Also, samples treated with PS showed significantly lower values of ALH than the C samples after submitting the samples to the incubation. Similarly, those samples treated with LS exhibited lower values (*P* < 0.05) of VSL respect to the control just after thawing. Finally, just after thawing, PS and G had significantly lower values of VAP (*P* < 0.05) than the C (Figure 1F). After the incubation, these significant differences disappeared.

### Membrane Integrity (Sperm Viability), Caspase 3 and 7 Activity, and Metabolic Activity

**Figure 2:**
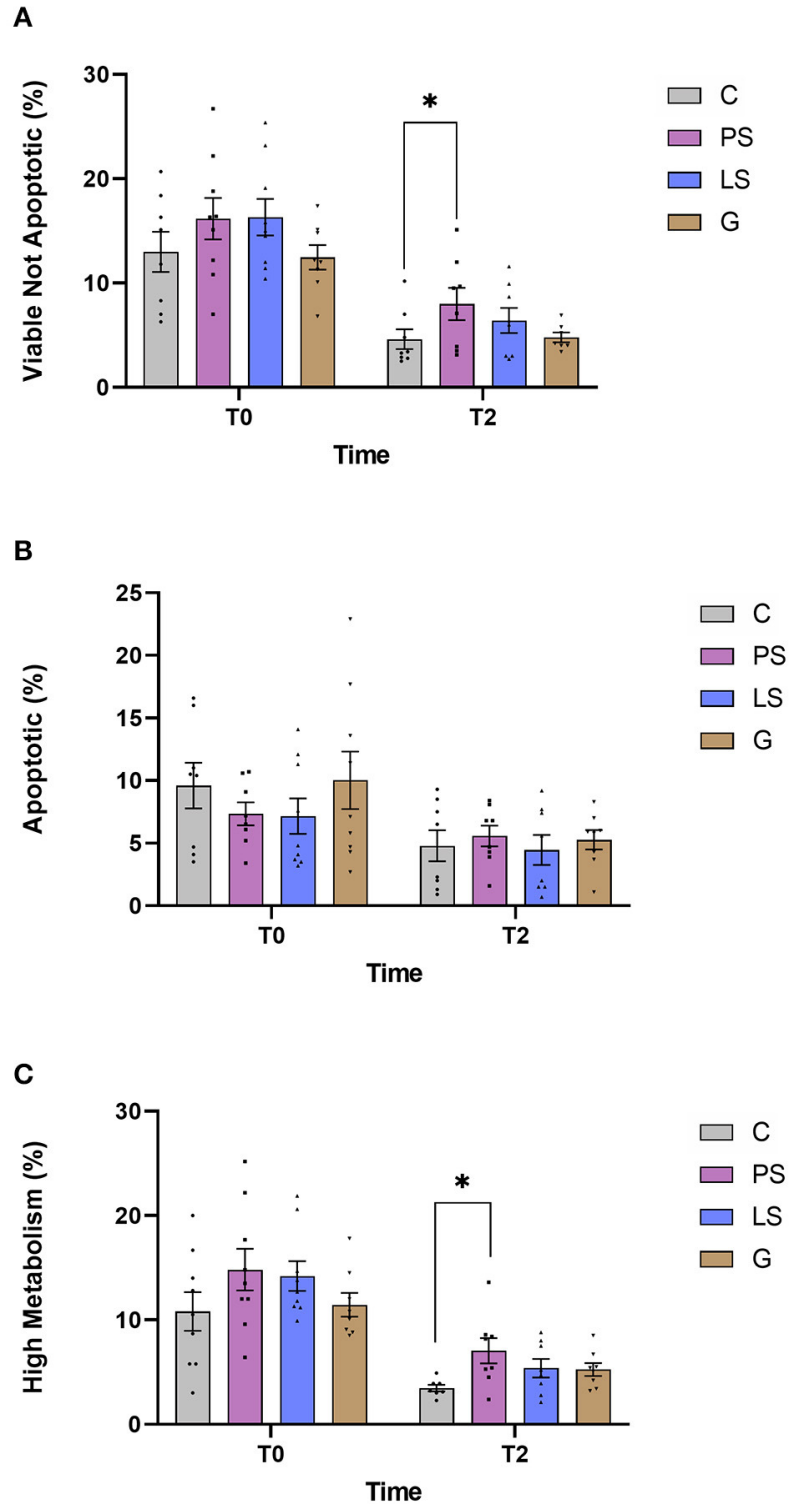
Ram sperm multiparametric flow cytometry data in the four experimental groups by time (just after thawing -T0- and after 2 h of incubation at 37°C -T2-): Control (C), without antibiotics; Penicillin-Streptomycin (PS); (500 UI)−625 μg/mL, respectively; Lincomycin-Spectinomycin (LS); 300–600 μg/mL, respectively; and Gentamicin (G) 1,000 μg/mL. **(A)** Zombie low intensity cells and Caspase 3&7 negative cells (viable not apoptotic sperm, %); **(B)** Zombie low intensity cells and Caspase 3/7 positive cells (apoptosis, %); **(C)** Zombie low intensity cells and CellROX-positive cells (Sperm with high metabolic activity, %). Nine males were analyzed (1 ejaculate per male) including the same males in each experimental group. Graph dots represent individual male ejaculate. Significant differences (*P* < 0.05) are represented with an asterisk between the antibiotic treatment and the Control sample without antibiotics.

The mean, standard error, and male distribution of the different evaluated sperm parameters are shown in Figure 2. No significant differences were found when assessing the viability just after thawing between the control and each treatment (Figure 2A). The viability of all groups was significantly lower (*P* < 0.05) after 2 h of incubation. After this incubation, PS kept higher values of non-apoptotic viable sperm than C (*P* < 0.05). Assessing the percentage of apoptotic cells, there were no significant differences among treatments either after thawing (T0) or after the incubation (T2) with respect to the C (Figure 2B). Finally, after assessing the metabolic status of sperm mitochondria via ROS generation, data indicated a higher tendency value (*P* < 0.06) in PS than the Control, not raising the significance, just after thawing. All treatments had a significant decrease (*P* < 0.05) after 2 h of incubation. However, PS showed significantly higher values of metabolic activity than the C after 2 h of incubation (*P* < 0.05). In contrast, LS and G did not show any significant differences (*P* > 0.05) compared to C (Figure 2C) for this parameter.

### Bacteriological Assessment

**Figure 3:**
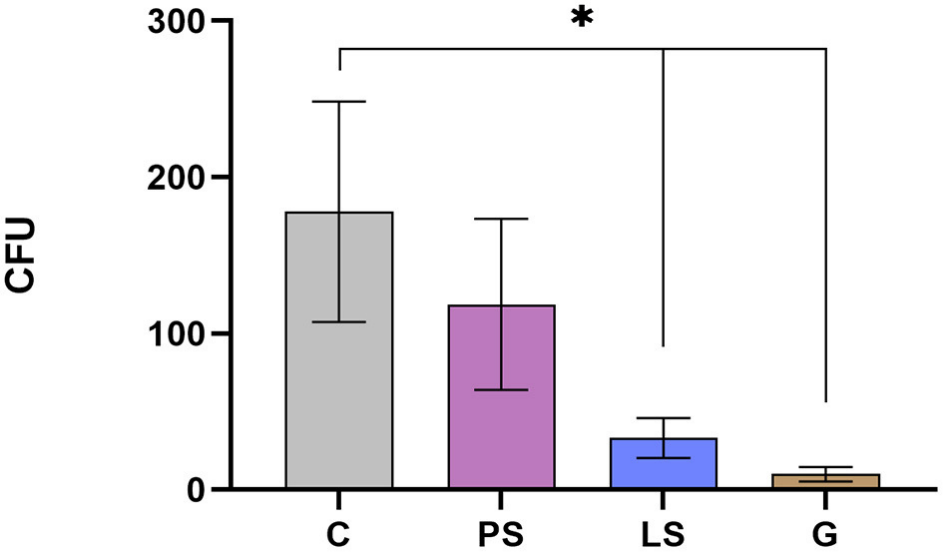
Total viable aerobic bacterial (colony-forming units) in the four experimental groups: Control (C), without antibiotics; Penicillin-Streptomycin (PS); (500 UI)−625 μg/mL, respectively; Lincomycin-Spectinomycin (LS) 300–600 μg/mL, respectively; and Gentamicin (G) 1,000 μg/mL. Eight males were analyzed (1 ejaculate per male) including the same males in each experimental group. Significant differences (*P* < 0.05) are represented with an asterisk between the antibiotic treatment and the Control sample without antibiotics.

The mean and standard error of the mean of CFU/mL recovered for each treatment is shown in Figure 3. A significant reduction in the number of viable bacteria after G and LS treatments as compared with the C without antibiotics was recorded (*P* < 0.05). PS treatment also decreases bacterial concentration although differences do not reach statistical significance when compared with control (*P* = 0.345; Figure 3).

**Figure 4:**
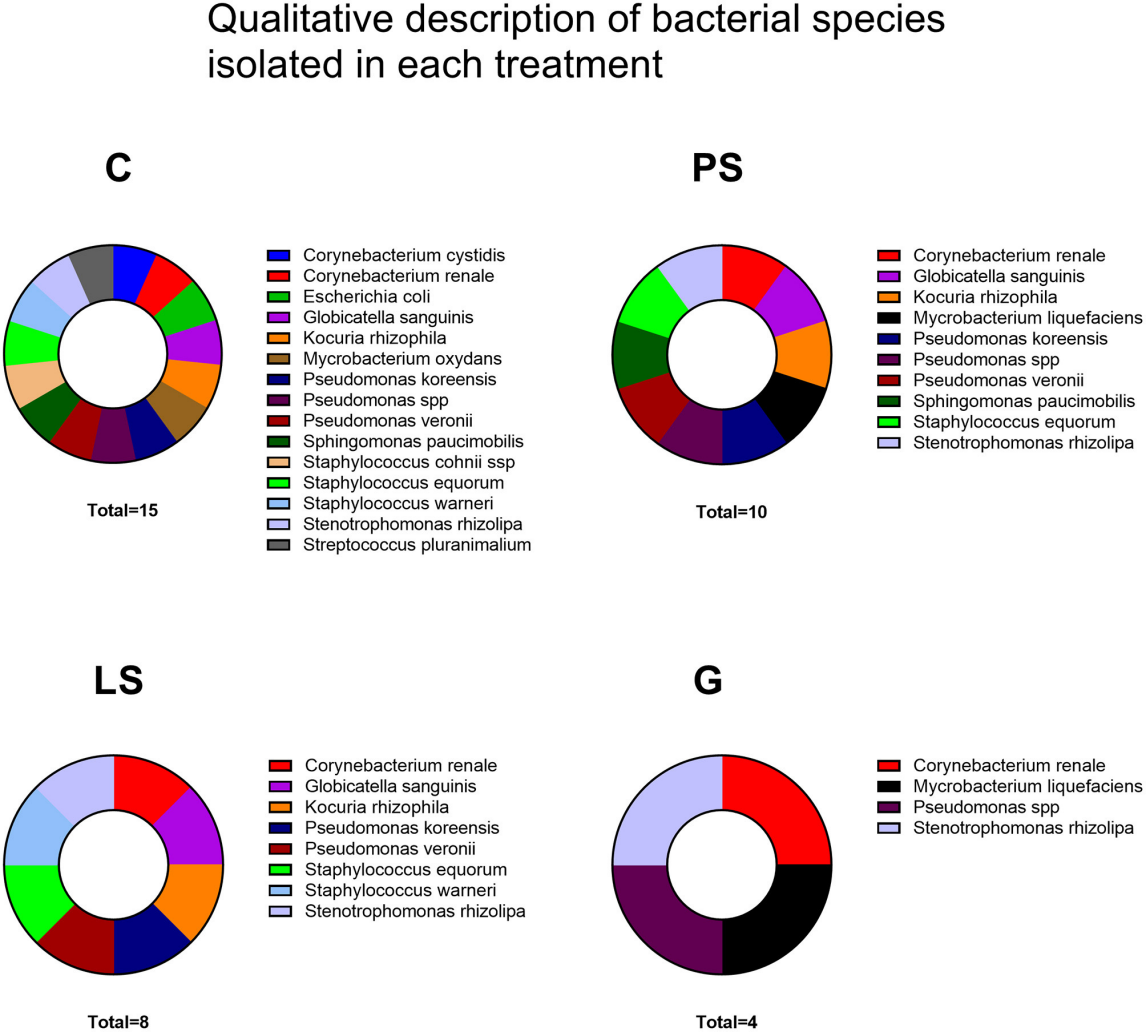
Bacterial species isolated in each treatment after thawing: Control (C), without antibiotics; Penicillin-Streptomycin (PS), (500 UI)−625 μg/mL, respectively; Lincomycin-Spectinomycin (LS), 300–600 μg/mL, respectively; and Gentamicin (G) 1,000 μg/mL. Eight males were analyzed (1 ejaculate per male) including the same males in each experimental group.

Most of the samples resulted in the growth of a mixed microorganism population with up to 16 different species. The highest number of different bacterial species was identified in the C group without antibiotics, followed by PS, LS, and finally G with the lowest number of different bacterial species (Figure 4). A total of 10 genera and 16 bacterial species (Figure 4) were identified with *Pseudomonas* (3 isolates) and *Staphylococcus* (3 isolates) as the most common genera followed by *E. coli*. In this sense, G was the treatment with the highest antimicrobial spectra (4 survivor species) followed by LS (8 survivor species) and PS (10 survivor species) (Figure 4).

### Fertility Trials

Samples treated with PS, the standard treatment, showed fertility of 52.0%. In the same way, samples treated with LS (50.5%) and C (52.9%) showed similar results (*P* > 0.05). In contrast, those samples treated with G exhibited the worst results (38.8%) being significantly lower (*P* < 0.05) than the other treatments (Table 1).

**Table 1:** Fertility results (lambed ewe/100 inseminated ewe) by treatment as follows: Control (without antibiotics), Penicillin-Streptomycin; with Penicillin (500 UI) and Streptomycin to a final concentration of 625 μg/mL; Lincomycin-Spectinomycin to a final concentration of 300 and 600 μg/mL, respectively; and Gentamicin to a final concentration of 1,000 μg/mL.

**Treatment**	**Inseminated**	**Lambed**	**Fertility (%)**
	**ewes (n)**	**ewes (n)**	
Control (without antibiotics)	221	117	52.9a
Penicillin-Streptomycin (500 UI−625 μg/mL)	211	105	49.7a
Lincomycin-Spectinomycin (300 and 600 μg/mL)	206	104	50.5a
Gentamicin (1,000 μg/mL)	214	83	38.8b

*Ten males were used*.

*Different low case letters (a,b) indicate significant differences (P < 0.05) among treatments*.

Similar prolificacy results were observed between C, G, and LS (*P* > 0.05), while PS (1.67 ± 0.07) showed significantly higher results (*P* < 0.05) than LS. Multiple lambing frequency followed exactly the same significance distribution among treatments as prolificacy (Table 2). The results obtained in fecundity showed one more time the lowest rate in those samples assessed with G (0.59±0.06), being significantly lower (*P* < 0.05) than the C and PS (0.81 ± 0.06 and 0.83 ± 0.07, respectively). For its part, samples treated with LS (0.74 ± 0.06) did not show significant differences with respect to the other treatments (Table 2).

**Table 2:** Multiple lambing frequency (%), prolificacy (lambs/lambed ewe), and fecundity results (lambs/inseminated ewes) by treatment as follows: Control (without antibiotics), Penicillin-Streptomycin; with Penicillin (500 UI) and Streptomycin to a final concentration of 625 μg/mL; Lincomycin-Spectinomycin to a final concentration of 300 and 600 μg/mL, respectively; and Gentamicin to a final concentration of 1,000 μg/mL.

**Treatment**	**Multiple lambing**	**Prolificacy**	**Fecundity**
	**frec. (%)**	**(MEAN±SE)**	**(MEAN±SE)**
Control (without antibiotics)	48.72ab	1.52 ± 0.05ab	0.81 ± 0.06a
Penicillin-Streptomycin (500 UI−625 μg/mL)	55.24a	1.67 ± 0.07a	0.83 ± 0.07a
Lincomycin-Spectinomycin (300 and 600 μg/mL)	41.35b	1.47 ± 0.06b	0.74 ± 0.06ab
Gentamicin (1,000 μg/mL)	45.78ab	1.53 ± 0.07ab	0.59 ± 0.06b

*Ten males were used*.

*Different low case letters (a,b) indicate significant differences (P < 0.05) among treatments*.

**Figure 5:**
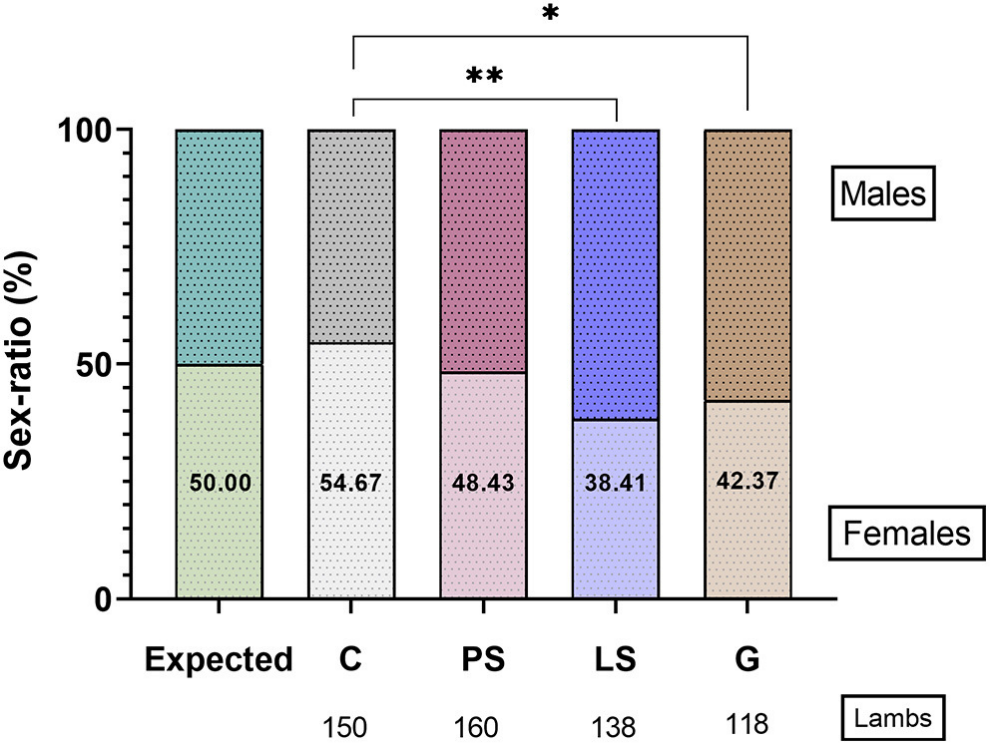
The sex-ratio results for female (females/100 lambs) in the four experimental groups: Control (C), without antibiotics; Penicillin-Streptomycin (PS), (500 UI)−625 μg/mL, respectively; Lincomycin-Spectinomycin (LS), 300–600 μg/mL, respectively; and Gentamicin (G) 1,000 μg/mL. 371 ewes were inseminated with sperm doses from 10 males. *P ≤ 0.05, **P ≤ 0.01.

There was no significant sex ratio distortion (*P* > 0.05) with the expected value (50:50) in each experimental group. The C obtained 54.7% of females, being the treatment with the highest deviation of sex-ratio to females (*P* < 0.05). On the opposite, the percentage of females at birth was lower (*P* < 0.05) for those samples treated with G (42.4%) and LS (38.4%) than C (Figure 5). The result obtained for the samples treated with PS was the closest to a expected proportion of 50% (48.4% females) and not being significantly different (*P* > 0.05) than the other 3 treatments (Figure 5).

## Discussion

The increasing challenge to health care attributable to antimicrobial resistance, and the subsequent absence of access to effective treatments, is a worldwide concern. In the same way, the use of antibiotics as an additive in sperm extender is a standard nowadays. Semen is normally colonized by a high variety of microorganisms that may reduce sperm quality. However, contradictory results on the effect of bacterial flora can be found, as well as in the effect of different antibiotic families depending on such important factors as the species, the sperm extender composition, or even the cryopreservation protocols (26). In this context, it is important to carry out an intensive assessment, not only assessing *in vitro* sperm parameters but also assessing important productive parameters, of the main antibiotic families used nowadays in sperm extenders and get a clear idea about their effect and the suitability of their use. In this study, a total of 10 genera and 16 bacterial species were identified with *Pseudomonas* and *Staphylococcus* as the most common genera followed by *E. coli*. Other bacterial species were also isolated in a lower number such as *Sphingomonas paucimobilis, Deftia acidovorans, Mycrobacterium oxydans, Kocuria*, and *Corynebacterium*. Some of these bacteria were also found by Yániz et al. (10) in ram sperm. In the same way, *E.coli*, which is one of the most common species isolated in the current experiment, has also been detected as the most frequent bacterial species in human (6), equine (18), canine (27), and porcine (20) sperm doses.

As we have previously described, the effect of antibiotics on sperm quality have controversial results attending to factors such as species, type of extender, or cryopreservation protocol. Many authors recommended some antibiotics such as gentamicin as a suitable additive for semen storage (18, 28, 29). In the present study, gentamicin was the antibiotic exhibiting the most powerful effect inhibiting bacterial growth together with LS. In contrast, Gentamicin was also the treatment with a lower sperm quality assessed *in vitro*. Jasko et al. (29) showed a negative effect on sperm motility when using gentamicin concentrations >1 mg/mL in equine sperm. On the contrary, Yániz et al. (10) found that the concentration of gentamicin in sperm extender up to 0.5 g/l did not affect sperm motility and viability after storage at 15°C for 48 h in ram. At this point, 1 g/L of gentamicin concentration looked like the safety limit for this type of antibiotic in equine. However, our results report a significant decrease in fertility and fecundity when this concentration of gentamicin was used. It could be possible that ram sperm are more sensitive to this type of antibiotic at this concentration than stallion. It should be taken into account that our study is the first time that a negative effect on fertility has been described in ram when gentamicin is used as an additive extender. With the same concentration (1 g/L) of Gentamicin, Aurich and Spergser (1) showed that sperm motility assessed *in vitro* was significantly reduced and the bacterial inhibition was not as high as expected in stallion sperm. This fact shows that there is a very important specie specific factor that can determine the efficiency of antibiotical substances in sperm extender among species. In this way, and after assessing our results, we can conclude that our concentration of gentamicin works greatly inhibiting the bacterial growth, but it resulted in a toxic effect on some sperm quality assesments (rapid progressive motility and different kinetic parameters: VAP, VSL, and ALH, Figure 2). This altered sperm quality could be affecting negatively important livestock parameters such as fertility. This fact is correlated to the decreased progressive motility assessed *in vitro*. In this context, high values of progressive motile sperm have been demonstrated as a good correlation factor with fertility in humans (30, 31). Probably this negative effect is due to some deleterious effects at the structural level since the sperm metabolism was not affected by the gentamicin respect to the C samples. A recent study carried out by Riesco et al. (32) demonstrated a novel sperm protein (ProAKAP4) as a promising diagnostic parameter of sperm quality in ram sperm, so long correlated to sperm motility. This protein is the most expressed protein of the sperm fibrous sheath being an important part of the flagellum (33, 34). This type of protein has also been related to sperm quality in several species such as humans (35), mice (36), or boar (37). In this sense, further investigations should be carried out to identify the exact mechanism of gentamicin toxicity. In any case, and in view of the results obtained in the present study, it is clear that gentamicin is not a suitable option, under these conditions, taking into account their negative effects, especially in the productive parameters.

The PS was the treatment with lower efficacy inhibiting bacterial growth. It gets a lower number of CFU but this decrease was not significant with respect to the control samples. This treatment (PS) has been one of the most common and extended antibiotics since it was discovered in 1928, so this intensive use for many decades could have produced a mechanism of resistance to this type of treatment (38, 39). In contrast, when the sperm samples were assessed *in vitro* those samples treated with PS showed the best results in the sperm physiology keeping higher values of viable sperm after submitting thawed samples to a stress test. The same effect was found when assessing the sperm with high mitochondrial activity, getting the best result of the experiment at both times 0 and 2 h. This fact could suggest that PS at this concentration is a suitable treatment since it inhibits in part the bacterial growth, not affecting the sperm quality. In the same way and after the field trials, PS keeps exhibiting similar results to Controls samples in fertility and prolificacy, not showing any adverse or toxic effect as the G in the fertility, or the LS in the prolificacy.

Finally, the LS treatment exhibited very interesting and complex results. After assessing sperm quality *in vitro*, LS did not show any deleterious effect either in the sperm physiology or in the sperm motility respect to the C samples. In addition, it was after the G, the treatment with the highest bacterial inhibitory activity, but not exhibiting adverse effects on the sperm quality *in vitro*. This fact could suggest that this treatment (LS) at this concentration has a perfect balance between the bacterial inhibitory effects, not affecting the sperm physiology in ram. In this study, similar results to Azawi and Ismaeel (16), when assessing sperm quality *in vitro*, were obtained but opposite results when assessing the effect inhibiting bacterial growth, finding these authors a poor antimicrobial effect using a concentration of 1 mg/ml of lincomycin in ram. These differences could be due to several factors such as the use of Lincomicyn alone (not combined with spectinomycin) or the experimental design (refrigerated samples at 5°C till 96 h). In any case, Lincomicyn seems to be more efficient when using combined with spectinomycin, allowing us to use lower concentrations, getting similar results in the sperm quality *in vitro*, and performing a very high inhibition in the bacterial growth. In the same way, Akhter et al. (19) have demonstrated the superiority of LS compared to PS combinations inhibiting bacterial growth in buffalo sperm samples. These and our results are in concordance with those obtained by Aleem et al. (40) in buffalo, where those authors agree that several bacterial species exhibited antimicrobial resistance to PS combinations while LS and others showed a higher antimicrobial effect. After the field trials, some interesting effects were observed. Neither fertility nor prolificacy or fecundity were affected as expected. However, after assessing the prolificacy we can observe that this parameter was lower in respect to the samples without antibiotic. This effect could be due to some kind of sublethal effect, not being capable to detect it *in vitro* as previously occurred in species such as boar (41) or buffalo (19). On the other hand, when the fecundity was assessed this lower prolificacy was compensated because the fecundity is a complex parameter where fertility and prolificacy are combined. This is the first time that these antibiotic treatments have been tested under field conditions in ram with a large number of ewes inseminated.

It is known that the plasma membrane from X or Y-sperm are diferent. Korchunjit et al. (42) found that different defined combinations of cryomedia and sperm extender significantly alter the survivar ratio of frozen-thawed X-Y sperm. In this sense, some interesting results were observed in our study when the sex ratio was assessed. Samples without antibiotics have deviated to females but LS and G had deviated to males. Neither the experimental group nor the control group has differences concerning the expected sex ratio (50:50). If we observed the results obtained in CFU and sex ratio we can observe that sperm samples with higher microbial charge result in a higher number of females, and the treatments with the lower microbial contamination (LS and G) resulted in a significantly lower number of females, which is negative in dairy species where females have the main economical value. At this point, it looks like X-sperm has some kind of resistance to microbial contamination, or that the Y-sperm are more resistant to some possible deleterious or toxic effect of LS and G. To this concern, these results pave the way for further investigations about the interaction bacteria/sperm, and to assess the different effect of antibiotic treatments in the different sperm subpopulations (Y or X-sperm).

The sex ratio is a complex parameter that depends on many biological and environmental factors. Preview studies have found both pre- and post-fertilization factors. Moreover, sex ratio bias may be related to both semen and the uterine environment in humans (43). Studies linking sperm quality and sex ratio are controversial. Some studies carried out in humans did not find differences in the sex ratio between seminal samples with a normal quality or with moderate or severe alterations (oligozoospermia, asthenozoospermia, and teratozoospermia) (44) or at least, this relationship is weak. However, in humans, Bae et al. (45) observed that the percentage of bicephalic sperm was significantly associated with the increase of born females. In the same way Arikawa et al. (46), using IVF, observed that samples with low sperm motility (<40%) had a minor proportion of males compared to those samples with normal sperm motility. This variation in the sex ratio (less males) observed with low-quality semen could be associated with a lower proportion of Y-bearing sperm in the ejaculate, as suggested by Eisenberg et al. (47) in humans. Contrary, in our study the samples with lower VAP, ALH, VSL, and rapid PM increase the percentage of born males according to Mossman et al. (48) in humans. Group G had fewer rapid progressive sperm *in vitro* and this could impair the transport of X-bearing sperm *in vivo*. A study conducted by Balli et al. (49) in humans showed an association of higher sperm velocity in semen from patients that conceived predominantly female offsprings when compared with patients with male offsprings after assisted reproductive technology (ART) treatment.

Finally, it could be interesting to do a deep reflection about the use of antibiotics as additives in sperm extenders. As mentioned above, the reduction of using antibiotics is a worldwide concern. Among the main causes of bacterial resistance, there are some social issues such as overpopulation or global migration, but also the increasing use of antibiotics in clinics and animal production (50, 51). Recent studies carried out in boar sperm (52) are trying to optimize sperm handling protocols to avoid or reduce the use of antibiotics. Taking into account the results obtained in the present study, when using healthy semen donors under satisfactory hygiene-pathology conditions and when the samples are going to follow a frozen-thawing process, non-deleterious effects have been observed either in the sperm quality assessed *in vitro* or in the productive parameters (fertility, prolificacy, or fecundity) when using sperm samples without antibiotics. Our results suggest that the bacterial contamination control in frozen-thawed semen may be possible without the use of antibiotics, although the effects of longer periods of cooling storage and different temperatures of storage need to be further investigated for animal semen.

## Data Availability Statement

The raw data supporting the conclusions of this article will be made available by the authors, without undue reservation.

## Ethics Statement

The current study was performed according to the Guidelines of the European Union Council (86/609/EU, modified by 2010/62/EU), following Spanish regulations (RD/1201/2005, abrogated by RD/2013) for the use of laboratory animals. All experimental protocols and procedures were approved by the Institutional Animal Care and Use Committee at the University of León (Spain) (ÉTICA-ULE-013-2018).

## Author Contributions

LA-L: conceptualization, methodology, formal analysis, investigation, resources, writing—original draft, data curation, writing—review & editing, visualization, supervision, and project administration. MR: methodology, investigation, supervision, data curation, formal analysis, and writing—review & editing. RM-G, MN-M, JB, CC, CO-F, and JA: methodology and investigation. AC: data curation, methodology, and investigation. PP: formal analysis, investigation, resources, writing—original draft, data curation, writing—review & editing, visualization, supervision, and funding acquisition. MA: conceptualization, methodology, investigation, resources, writing—original draft, data curation, writing–review & editing, visualization, and funding acquisition. LA: conceptualization, resources, formal analysis, resources, writing—original draft, data curation, writing—review & editing, visualization, supervision, project administration, and funding acquisition. All authors contributed to the article and approved the submitted version.

## Conflict of Interest

The authors declare that the research was conducted in the absence of any commercial or financial relationships that could be construed as a potential conflict of interest.
